# Pollen sensitization profiles of allergic patients in a middle European region

**DOI:** 10.1186/1939-4551-8-S1-A153

**Published:** 2015-04-08

**Authors:** Petr Panzner, Martina Vachova, Petra Vitovcova, Tomas Vlas

**Affiliations:** 1 Faculty of Medicine and Faculty Hospital in Pilsen, Czech Republic

## Background

The aim of our study was to assess the pollen sensitization patterns by means of molecular diagnosis approach in the region of Pilsen, Czech Republic.

## Methods

The microarray system ImmunoCAP ISAC has been used for specific IgE detection to 113 different allergenic molecules. Sera from 989 patients sensitized to at least one pollen-derived molecule were subject of analysis. These patients suffered from at least one of the following diagnoses: chronic rhinitis (63%), bronchial asthma (33%), atopic dermatitis (29%), urticaria or angioedema (26%) and/or anaphylaxis (10%). Patient age ranged from 2 to 68 years, with a mean age of 32,6 years. The sex ratio was 39,0% men to 61,0% women.

## Results

The most frequent sensitization rate was observed to grass-derived species specific molecules (81.2% overall), the most frequent being Phl p 1 (65.6%), markedly overwhelming sensitization rates to any non-pollen-derived molecule. The second one were pollen-derived PR-10 molecules (52.2% overall), of which the large majority included Bet v 1 (51.9%). Sensitization to these two types of pollen components (and their co-sensitizations with other components) forms the vast majority of pollen sensitizations. The patterns of co-sensitization is presented by means of Venn diagram approach. Sensitization to Cupressaceae-derived molecules was observed in 15.1% of subjects, to Oleaceae derived-molecules in 12.3% (Ole e 1 and Ole e 9 in 8.8% and 3.5% resp.) and to the plane tree-derived molecules Pla a 2 and Pla a 3 in 14.2% and 3.5% resp; these relatively high rates were surprising as the respective pollens have not been considered as important in the region. The sensitization rates for further molecules were: Art v 1 – 13.2%, Pla l 1 – 10.8%, Che a 1 - 9.6%, Par j 2 – 1.1%, Sal k 1 – 0.4% and Amb a 1 – 0.9%. The sensitization rates to cross-reacting molecules were generally not as high as reported from other regions (profilins 12.4%, polcalcins 5.0%, LTPs 6.4%). Only 1.8% patients reacted to pollen-derived panallergen and not simultaneously to a pollen species-specific component.

## Conclusions

Molecular diagnosis of allergy gives a more precise and comprehensive insight into pollen sensitization patterns than extract-based testing, allowing for better understanding of the sensitization process and regional differences. The data presented may help to improve diagnostic and treatment specific procedures in the respective region.

